# Scalable and Privacy-Conscious End-to-End Processing of Large-Scale Clinical Data for Precision Medicine: Empirical Evaluation Study

**DOI:** 10.2196/83487

**Published:** 2026-03-04

**Authors:** Jungwoo Lee, Sangwon Hwang, Kyu Hee Lee

**Affiliations:** 1 Artificial Intelligence Big Data Medical Center Yonsei University Wonju College of Medicine Wonju Republic of Korea; 2 Department of Precision Medicine Yonsei University Wonju College of Medicine Wonju Republic of Korea

**Keywords:** electronic health records, medical informatics, predictive performance, data privacy, precision medicine

## Abstract

**Background:**

In large-scale clinical data analysis, CSV and traditional relational database management system–based approaches are widely used but impose substantial storage and processing constraints that delay research preparation and hinder multicenter collaboration. Although column-oriented storage formats such as Apache Parquet have gained attention in data science, systematic end-to-end evaluations in clinical environments remain limited, particularly regarding efficiency and scalability.

**Objective:**

This study aimed to empirically evaluate whether a Parquet-based end-to-end pipeline could improve computational efficiency and scalability in large-scale clinical data analysis while preserving predictive performance and protecting privacy.

**Methods:**

Electronic health record data comprising 13.76 million rows from a large academic medical center in Korea were analyzed using Parquet, CSV, PostgreSQL, and DuckDB environments. Standardized SQL workloads and multilabel classification models—implemented using graphics processing unit–accelerated Extreme Gradient Boosting and classifier chain (CC) ensembles to address class imbalance—were applied to evaluate storage efficiency, time to analysis, and predictive performance. Statistical equivalence testing with prespecified clinical margins and bootstrap resampling ensured rigorous comparison, while privacy risks were assessed through advanced membership inference attacks (MIA), including shadow MIA and likelihood ratio attacks.

**Results:**

Compared with CSV, Parquet demonstrated enhanced computational efficiency by lowering disk access from 940.2 to 44.2 seconds (95.3% reduction). End-to-end processing latency was substantially reduced across feature transformation (15.0 vs 9.3 s) and model training (8.1 vs 6.7 s). To address complex clinical correlations, we implemented CC and one-vs-rest architectures, which effectively captured interdependencies between disease labels. Classification performance remained statistically equivalent across area under the receiver operating characteristic curve, area under the precision-recall curve, accuracy, and *F*_1_-score, with all differences falling within prespecified clinical equivalence margins (*P*<.001). Notably, the CC ensemble demonstrated high technical rigor, minimizing Hamming loss (2.2×10^–4^) and ensuring robustness even in imbalanced cohorts. MIA performed at chance level (area under the curve=0.500), suggesting no measurable increase in privacy risk.

**Conclusions:**

By significantly mitigating data processing bottlenecks, a Parquet-based pipeline enabled high-throughput, large-scale clinical evidence generation without compromising model integrity or patient privacy. This framework provides a scalable and robust infrastructure for precision medicine, facilitating agile multicenter collaborations and real-world data analysis in resource-constrained clinical environments.

## Introduction

Clinical data, including diagnostic reports, prescriptions, laboratory results, and imaging data, hold substantial potential for precision medicine, predictive modeling, and collaborative research. The volume of electronic health records (EHRs) generated in clinical practice has grown exponentially with the expansion of digital health systems [1-4]. However, as datasets expand, bottlenecks, such as storage overhead, transmission delays, and slow query execution, become increasingly common. These challenges cannot be resolved simply by expanding storage capacity; instead, they necessitate more efficient frameworks for data storage and processing.

In medical informatics, data are most commonly stored in row-oriented formats, such as CSV files, spreadsheets, and relational databases. Although valued for their simplicity and interoperability, these approaches exhibit clear limitations in storage efficiency and analytic scalability. CSV files are uncompressed and large in size, restricting input and output throughput, while relational databases often underperform in column-level operations. In contrast, column-oriented formats offer high compression and selective input and output, making them well suited for high-volume and cloud-based environments. Among these, Apache Parquet is widely used as a standard solution owing to its scalability and interoperability [5,6]. However, few studies have systematically evaluated its end-to-end performance in clinical pipelines that incorporate deidentification procedures.

A further challenge in clinical data use is balancing privacy protection with data utility. Deidentification is mandatory in multicenter research and machine learning, but it may affect statistical distributions or compromise predictive performance. Understanding how storage architectures interact with deidentification to influence analytic outcomes is therefore of both academic and practical importance [7-13].

Unlike prior studies that focused solely on benchmarking computational efficiency, this work integrates privacy risk assessment and formal equivalence testing into a real-world end-to-end pipeline, thereby providing a clinically meaningful evaluation of Parquet as a foundation for precision medicine and collaborative research. Against this background, this study developed a scalable Parquet-based pipeline for high-volume clinical data and empirically assessed its storage efficiency, computational throughput, and preservation of predictive performance. This study also sought to establish a reliable infrastructure that enables cohort construction, secure data sharing, and clinical decision support in multicenter and precision medicine research [14-23].

## Methods

### Ethical Considerations

This retrospective observational study was conducted using real-world EHR data. The study protocol was approved by the institutional review board of Wonju Severance Christian Hospital (CR325065). The requirement for informed consent was waived by the institutional review board due to the retrospective nature of the study and the use of deidentified data. All data processing was performed within an independent honest broker system, and investigators had access only to deidentified data. No compensation was provided to the participants as there was no direct contact or intervention involved. The entire process complied with the Personal Information Protection Act of Korea, the Health Insurance Portability and Accountability Act Safe Harbor standard, and the General Data Protection Regulation [24-28].

### Integrated End-to-End Clinical Data Processing Workflow

**Figure 1:**
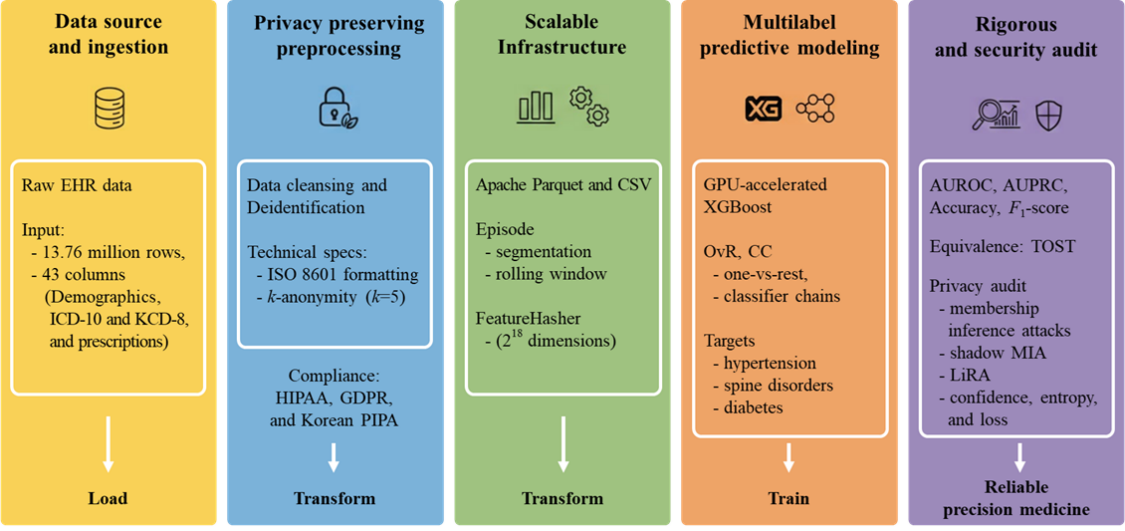
Integrated workflow for scalable and privacy-preserving clinical data processing. AUROC: area under the receiver operating characteristic curve; AUPRC: area under the precision-recall curve; CC: classifier chains; EHR: electronic health record; GDPR: General Data Protection Regulation; GPU: graphics processing unit; HIPAA: Health Insurance Portability and Accountability Act; ICD-10: International Classification of Diseases, Tenth Revision; KCD: Korean Classification of Diseases, Eighth Revision; LiRA: Likelihood Ratio Attack; MIA: membership inference attack; OvR: one-vs-rest; PIPA: Personal Information Protection Act; TOST: two 1-sided tests; XGBoost: extreme gradient boosting.

We developed an integrated pipeline connecting 5 core stages: raw EHR ingestion, privacy-preserving preprocessing (ISO 8601 and *k*-anonymity), scalable infrastructure using Apache Parquet, multilabel modeling via graphics processing unit (GPU)–accelerated Extreme Gradient Boosting (XGBoost), and rigorous statistical and privacy audits (Figure 1). To optimize analytic performance, we adopted a column-oriented storage format (Apache Parquet) over the traditional row-oriented structure (eg, CSV) [6]. For comparative evaluation, the same dataset was also maintained in CSV format. While row-oriented structures excel in transactional tasks, columnar storage significantly reduces input and output overhead and enhances compression efficiency for complex analytic queries. This architectural choice is specifically tailored for the high dimensionality and sparse distribution of clinical datasets, ensuring both system scalability and robust privacy protection across the entire workflow.

### Data Preparation and Transformation

The dataset included patient demographics, diagnostic codes based on the International Classification of Diseases, Tenth Revision (ICD-10) [29-35] and the Korean Classification of Diseases, Eighth Revision (KCD-8), visit type, and prescription records. In total, the dataset comprised approximately 13.76 million rows (43 columns, approximately 3.2 GB), as detailed in Multimedia Appendix 1. After replacing patient identifiers with randomly generated surrogate IDs, quasi-identifiers were generalized to satisfy *k*-anonymity (*k*=5). Data cleansing further ensured quality control by standardizing date fields into the ISO-compliant format (YYYY-MM-DD).

### Clinical Data Processing Pipeline

**Figure 2:**
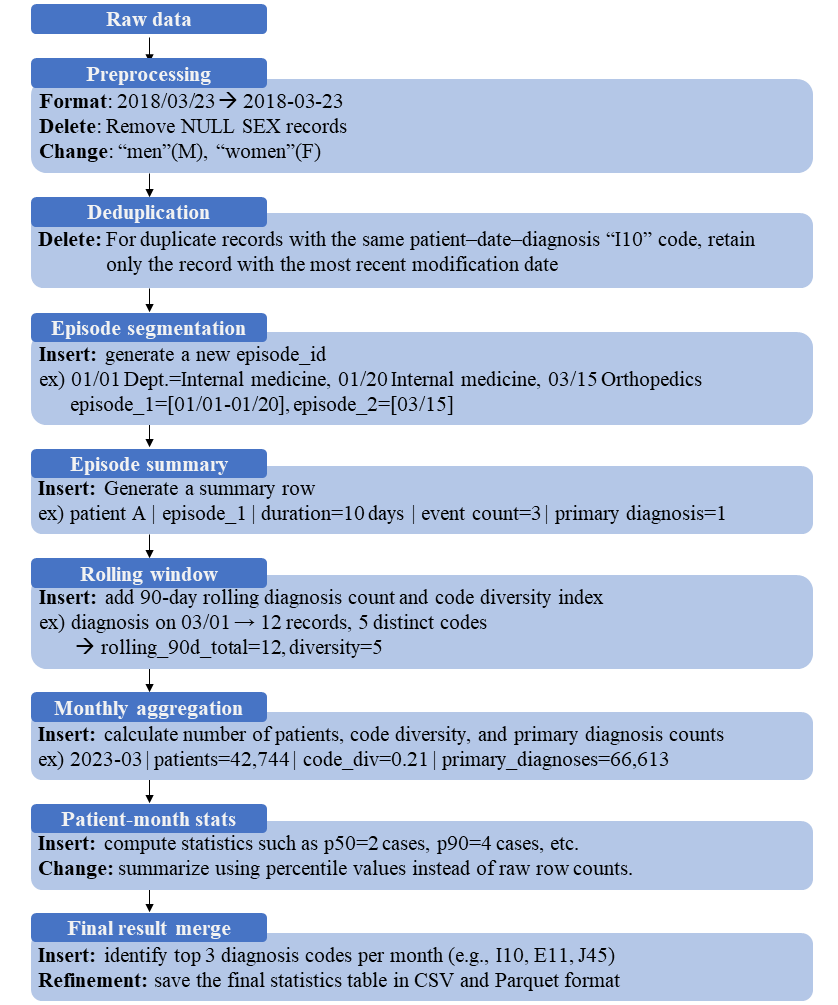
Stepwise scenario of the clinical data processing pipeline.

Clinical data processing followed a stepwise pipeline designed to reflect clinical context. In preprocessing, we standardized date formats, removed records with missing sex, and normalized sex codes (eg, “men” to M and “women” to F). Duplicate entries were resolved by retaining only the most recent record for identical combinations of patient, date, and diagnosis code. Visits were segmented into episodes by department changes or temporal gaps, and each episode was assigned an episode identifier. Episode-level summaries included episode duration, event count, and primary diagnosis status. To capture temporal continuity, a 90-day rolling window was applied to compute diagnosis counts and code diversity per patient. Monthly aggregates were then derived, including patient counts, code diversity, and primary diagnosis counts, with patient-month statistics summarized using quantiles (eg, p50 and p90). The diagnostic records for the targeted conditions—including I10.9, E10-E14, and M48.06—were identified and extracted to construct the final summary tables, which were then stored in both CSV and Parquet formats for comparative analysis. The stepwise process is illustrated in Figure 2.

### Database Workload Benchmarking

To evaluate the efficiency of storage formats, scenario-based workloads were executed in DuckDB (DuckDB Labs) [36] and PostgreSQL (PostgreSQL Global Development Group) [37] environments. Synthetic example data are provided in Multimedia Appendix 2. Identical datasets were loaded into Parquet-based DuckDB and row-store PostgreSQL, and complex SQL queries—adapted from TPC-DS patterns [38,39] (eg, window functions, multidimensional aggregation, and ROLLUP)—were applied to compare performance. Resource use was further examined by varying chunk sizes (10,000-50,000 records) with detailed results in Multimedia Appendix 3.

### Predictive Modeling and Multilabel Strategy

Predictive modeling assessed the impact of storage formats on classification performance for hypertension (I10.9), spinal disorders (M48.06), and diabetes (E10-E14). Patient-level diagnostic histories were transformed into 2^18^ dimensional sparse vectors (approximately 262,000 features) via FeatureHasher (scikit-learn; Python Software Foundation) [40-42]. The dataset was randomly partitioned into training (65%), validation (15%), and test (20%) subsets with a fixed seed of 42. Classification models were implemented using GPU-accelerated XGBoost with positive class weighting to address imbalance [43-45]. Hyperparameter details are provided in Multimedia Appendix 4.

For multilabel classification, we applied one-vs-rest (OvR) [46] and classifier chains (CC) [47]. To mitigate label-order dependence and evaluate interlabel dependencies, we constructed ensembles of 5 strategically ordered chains encompassing a prevalence gradient—from high-prevalence anchors (hypertension and diabetes) to the sparse target, spinal stenosis (M48.06, 0.50%; Multimedia Appendix 5). Prioritizing M48.06, a domain-independent and sparse condition, served to demonstrate model robustness even without the clustering benefits of related disease families (eg, I-series codes). To ensure the rigorous prevention of label leakage and validate the integrity of the performance gains, all defining ICD-10 codes were strictly excluded from the feature space. This setup ensured that the model’s predictive capability relied solely on statistical associations across hundreds of thousands of nondiagnostic features. Model performance was evaluated using area under the receiver operating characteristic curve (AUROC), area under the precision-recall curve (AUPRC), accuracy, and *F*_1_-score, calculated for each label as well as macroaverages and microaverages.

### Statistical Evaluation and Privacy Assessment

Model performance was evaluated using AUROC, AUPRC, accuracy, and *F*_1_-score. To assess model reliability, calibration was further evaluated using the Brier score and expected calibration error (ECE), with detailed results provided in Multimedia Appendix 6. CSV and Parquet results were compared using paired runs with shared identifiers. Normality was assessed via the Shapiro-Wilk test, followed by paired 2-tailed *t* tests or Wilcoxon signed-rank tests with Bonferroni correction (*P*<.05) [48-50].

Equivalence was assessed via two 1-sided tests (TOST) [51] and 95% bootstrap CIs. Equivalence margins (δ) were prespecified at 0.02 for AUROC or AUPRC and 0.01 for accuracy or *F*_1_-score. These thresholds were selected to be narrower than the 0.05 margin commonly used in clinical equivalence trials [52] and are more conservative than performance variances typically observed in the external validation of clinical artificial intelligence (AI) systems [53]. Such strict margins were intended to constrain observed differences to ranges generally regarded as clinically negligible, particularly for high-performing models operating within an elevated accuracy range (0.98-1.00).

To mitigate stochastic variation, a fixed random seed was applied across all experiments, ensuring that observed differences reflected data handling effects rather than algorithmic randomness. Privacy was further audited using membership inference attacks (MIA)—including confidence, entropy, loss-based (Yeom), shadow, and Likelihood Ratio Attack (LiRA) strategies—under patient-level stratified splits (train: 240,596; test: 240,597) to verify model robustness.

### Experimental Setup and Reproducibility

All experiments were conducted on a Linux Ubuntu 22.04 server with a 24-core central processing unit (CPU), 128-GB memory, and an NVIDIA RTX 5090 GPU (CUDA 12.2, cuDNN 8.9, driver 550.54). Data processing used Python (version 3.10; Python Software Foundation) with Pandas 1.5.3, PyArrow 12.0.1, scikit-learn 1.3.2, and XGBoost 2.0.3 (GPU-hist). Multiprocessing was implemented using the standard Python multiprocessing module.

As specified in the modeling strategy, XGBoost was configured with a learning rate of 0.08, maximum depth of 6, and 1000 estimators. To handle class imbalance, task-specific *scale_pos_weight* and an ensemble of 5 CCs were used. All random operations used a fixed seed of 42 for reproducibility. For robust performance evaluation, we conducted 20 independent experimental runs and used 5000-iteration bootstrap resampling to compute 95% CIs. Detailed hyperparameters and the statistical framework for equivalence testing are provided in Multimedia Appendix 4.

## Results

### Storage and Pipeline Efficiency

Conversion to Parquet substantially improved storage efficiency, reducing file size by nearly 6.9-fold relative to CSV. As summarized in Table 1, Parquet also consistently outperformed CSV across the end-to-end pipeline. File loading (input and output) time decreased by more than 95%, and both feature transformation (FeatureHasher) and model training (XGBoost) were faster under Parquet. Although peak CPU memory was about 1.3 GB higher, GPU memory consumption decreased by 326 MB, resulting in stable overall resource use. Resource consumption remained stable at 9 to 10 GB across chunk sizes, reflecting consistent scalability, with efficiency varying by chunk size. The values in Table 1 represent peak use in end-to-end runs, with minor variations arising from measurement conditions, whereas CPU, memory, and disk input and output patterns remained consistent across chunk sizes (Multimedia Appendix 3).

**Table 1:** System performance comparison between CSV and Parquet formats.

Metric			CSV, mean (SD)		Parquet, mean (SD)		Difference^a^ (95% CI)
**Time (s)**							
	Input and output duration	940.2 (53.3)		44.2 (1.6)		−896.0 (−930.0 to −860.0)	
	Feature transformation	15.0 (0.9)		9.3 (0.7)		−5.7 (−7.0 to −4.5)	
	Model training	8.1 (1.2)		6.7 (0.9)		−1.4 (−2.5 to −0.3)	
**Resource use**							
	Memory maximum (GB)	21.2 (0.8)		22.5 (0.6)		1.3 (0.1 to 2.0)	
	GPU^b^ memory maximum (MB)	4873.0 (582.6)		4547.0 (596.9)		−326.0 (−450.0 to −200.0)	

^a^Calculated as the Parquet mean minus CSV mean.

^b^GPU: graphics processing unit.

### Classification Performance and Calibration

Single-label results are presented in Table 2. All 3 tasks achieved AUROC values above 0.920, with AUPRC ranging from 0.488 to 0.580. Hypertension (I10.9) yielded the highest *F*_1_-score (0.599), followed by spinal disorders (M48.06, 0.536) and diabetes (E10-E14, 0.521). Macroaveraged performance reached an AUROC of 0.920, an AUPRC of 0.545, an accuracy of 0.934, and an *F*_1_-score of 0.552. Calibration analyses [54,55], reported in Multimedia Appendix 6, demonstrated excellent reliability for hypertension (Brier score 2.1×10^–5^; ECE 6.0×10^–6^), while spinal disorders (ECE=0.016) and diabetes (ECE=0.018) showed mild miscalibration but remained within recommended thresholds (ECE<0.020).

**Table 2:** Per-label classification performance using extreme gradient boosting.

Labels	AUROC^a^	AUPRC^b^	Accuracy	*F*_1_-score
Hypertension	0.920	0.580	0.906	0.599
Spine disorder	0.941	0.488	0.982	0.536
Diabetes^c^	0.899	0.567	0.915	0.521
Macroaverage	0.920	0.545	0.934	0.552

^a^AUROC: area under the receiver operating characteristic curve.

^b^AUPRC: area under the precision-recall curve.

^c^Diabetes mellitus (International Classification of Diseases, Tenth Revision codes: E10-E14).

Table 2 evaluates label-specific discrimination under substantial class imbalance, whereas Table 3 summarizes overall multilabel prediction performance using microaveraged aggregation across all label–instance pairs. Specifically, Table 2 focuses on the predictive performance of individual sparse labels, while Table 3 reflects aggregated performance across all prediction events. Multilabel results in Table 3 confirmed high-performance consistency for both OvR and CC, with AUROC >0.999 and AUPRC >0.998. Subset accuracy was identical (0.999), but CC showed slight improvements in Hamming loss and Jaccard index [56], suggesting modest benefits from capturing label dependencies. A numerical gap was observed between per-label AUPRC (approximately 0.5; Table 2) and multilabel AUPRC (near 1.0; Table 3). This reflects the technical difference in aggregation: multilabel metrics were calculated using microaveraging to evaluate the overall model efficacy.

**Table 3:** Multilabel classification performance.

Models			AUROC^a^		AUPRC^b^		Hamming loss		Subset accuracy		Jaccard index
**One-vs-rest**											
	Microaverage	0.999		0.998		2.4×10^–4^		0.999		0.997	
	Macroaverage	0.999		0.998		—^c^		—		—	
**Classifier chains**											
	Microaverage	0.999		0.998		2.2×10^–4^		0.999		0.997	
	Macroaverage	0.999		0.998		—		—		—	

^a^AUROC: area under the receiver operating characteristic curve.

^b^AUPRC: area under the precision-recall curve.

^c^Not applicable.

Although discrimination remained high across all tasks, calibration analyses revealed modest overconfidence for diabetes and spinal disorder models. These deviations, while within accepted thresholds, emphasize the importance of calibration-aware adjustments when deploying predictive models in clinical workflows.

### Database Benchmarking

As summarized in Table 4, DuckDB executed analytic workloads with an approximately 28-fold reduction in wall-clock time compared with PostgreSQL, which required 2546.4 seconds. DuckDB also exhibited higher CPU use (72.1% vs 39.6%), while memory consumption was comparable across systems.

**Table 4:** Performance comparison of DuckDB and PostgreSQL in scenario-based SQL workloads.

Metrics	DuckDB^a^	PostgreSQL^b^
Wall-clock time (s)	90.2	2546.4
CPU^c^ use (%)	72.1	39.6
Memory use (GB)	13.7	14.8
Read throughput (MB/s)	554.7	365.2
Write throughput (MB/s)	1116.0	998.0

^a^DuckDB: column-oriented database optimized for analytical workloads.

^b^PostgreSQL: row-oriented relational database primarily designed for transactional workloads, with support for analytical queries.

^c^CPU: central processing unit.

### Equivalence Analysis

Paired experiments (20 runs per condition) were conducted using shared run identifiers, with median and IQR values provided in Table S1 in Multimedia Appendix 7. Several metrics converged across all repetitions, producing zero IQR values and demonstrating high algorithmic stability. Overall, both formats achieved robust numerical consistency across all experimental iterations; for instance, mean AUROC was 1.000 for CSV and 0.984 for Parquet (Δ=−0.015). While other metrics (AUPRC, accuracy, and *F*_1_-score) were slightly higher under the CSV format, paired *t* tests and Wilcoxon signed-rank tests confirmed statistical significance after Bonferroni correction (*P*<.001; Table S2 in Multimedia Appendix 7). However, these numerical variations remained minimal.

Notably, all observed differences fell within the prespecified equivalence margins (δ=0.02 for AUROC and AUPRC and δ=0.01 for accuracy and *F*_1_-score). TOST confirmed statistical equivalence (*P*<.05) across all evaluated metrics, and bootstrap 95% CIs for AUROC Δ (−0.016 to −0.015) further reflected near-zero variability consistent with asymptotic performance stability (Table S3 in Multimedia Appendix 7).

### Privacy Assessment

The MIA evaluations, performed based on established methodologies [57-59] and summarized in Table 5, produced area under the curve (AUC) values of 0.501, 0.502, and 0.499 for confidence, entropy, and loss-based strategies, respectively. The maximum advantage did not exceed 0.007, and the gain over random guessing was ≤0.003. Attack performance was indistinguishable from random guessing, indicating no additional privacy risk. Additional shadow and LiRA [60,61] results are available in Multimedia Appendix 8, corroborating these findings.

**Table 5:** Membership inference attack results across different attack strategies.

Attack strategy	Attack AUC^a^ (95% CI)	Advantage	Best accuracy	Gain vs baseline
Confidence	0.501 (0.501-0.503)	0.006	0.503	0.003
Entropy	0.502 (0.502-0.503)	0.007	0.503	0.003
Loss (Yeom)	0.499 (0.498-0.499)	0.002	0.500	0.001

^a^AUC: area under the curve.

## Discussion

### Principal Findings

This study successfully implemented an integrated end-to-end pipeline (Figure 1) that ensures a seamless transition from raw EHR data ingestion to predictive modeling. By leveraging Apache Parquet, the framework effectively addressed storage and processing bottlenecks—specifically, input and output latency—inherent in traditional row-oriented workflows, substantially enhancing data throughput and accelerating the analytic preparatory phase while maintaining high predictive fidelity. Furthermore, our findings indicate that the minor performance discrepancies between formats are attributable to systematic data handling effects under a strictly controlled environment. Beyond these computational efficiencies, privacy evaluations demonstrated that the optimized workflow maintains rigorous data protection standards, providing a secure foundation for multicenter research operations without introducing additional vulnerabilities.

Although paired statistical tests yielded highly significant *P* values (*P*<.001), this result reflects the sensitivity of paired testing under large-scale data and repeated bootstrap evaluation rather than clinically meaningful divergence. From an equivalence perspective, all performance differences remained well within the prespecified margins, confirming that the Parquet-based pipeline achieves practical performance equivalence to the traditional CSV workflow.

The substantial improvement in multilabel AUPRC compared with per-label performance highlights the system-level predictive advantage gained by explicitly modeling interlabel dependencies. While individual disease prediction remains challenging under substantial class imbalance, the multilabel formulation enables the model to capture clinically meaningful comorbidity structures, thereby reframing the observed performance gap as a reflection of holistic predictive efficacy rather than a metric artifact. This gap was also driven by the extreme class imbalance inherent in clinical datasets (eg, 0.50% prevalence for spinal disorders), where high AUROCs can coexist with lower *F*_1_-scores. By integrating these dependencies within the CC architecture, the model leveraged frequent clinical co-occurrence patterns—such as hypertension and diabetes—to stabilize predictions for sparse conditions, including spinal stenosis. Importantly, the consistent performance observed despite the strict exclusion of all defining ICD-10 codes provided strong evidence against label leakage and underscored the model’s reliance on nondiagnostic clinical associations.

### Clinical Implications and Research Operations

The clinical implications were considerable. By substantially shortening preparatory phases and enabling analyses to be completed within hours rather than days, Parquet-based pipelines allowed investigators to progress more rapidly from raw data to actionable evidence. Such improvements not only accelerated study initiation but also facilitated the integration of predictive models into research and clinical workflows. In multicenter settings, scalability supported the efficient integration of heterogeneous patient populations without excessive computational cost, thereby enhancing reproducibility and advancing precision medicine initiatives. These efficiencies were directly relevant to clinical workflows and regulatory-grade analyses. They enabled interim analyses and timely regulatory submissions, supporting more responsive and efficient research operations.

### Privacy and Security Safeguards

Privacy evaluation reinforced the suitability of this approach. Deidentification procedures satisfied k-anonymity, and MIA yielded chance-level performance. The chance-level performance of multiple MIA methods confirmed that computational gains do not compromise patient confidentiality, a prerequisite for secure collaboration across institutions. Patient-level stratification and consistent pipeline design acted as effective safeguards. While residual risks remain inherent to secondary data use, our findings suggested that Parquet does not introduce additional vulnerabilities. For collaborative research networks—where privacy assurances are essential for sharing—these results provide practical reassurance.

### Interpretation of Predictive Performance and Label Dependencies

Classification analyses revealed stable performance for common conditions such as hypertension, whereas imbalanced disorders such as diabetes and spinal disease yielded lower scores. This pattern highlights the need to account for dataset structure and interlabel dependencies when interpreting predictive outputs. The elevated AUROC values likely reflect the combination of high-prevalence conditions and extensive diagnostic feature sets. Consistent with the modeling strategy, defining codes were excluded from the feature space; however, correlated diagnostic features may still have influenced performance estimates.

While performance convergence at elevated levels constrained the sensitivity to subtle comparative differences, it underscored the robustness and reproducibility of the pipeline, supporting its suitability for large-scale clinical research, where stability and reliability are critical. Predictive reliability was not compromised by storage structure, supporting Parquet as a dependable option for secondary use of EHR data in diverse research settings.

### Model Reliability and Calibration

Calibration analyses further showed that hypertension models were well aligned with observed risks, whereas diabetes and spinal disorder tasks exhibited modest miscalibration. These results underscore that even high-performing classifiers may generate overconfident probability estimates, a limitation directly relevant to clinical decision support. Future studies should evaluate calibration-aware techniques, such as temperature scaling or isotonic regression, to improve the reliability of probability estimates (Multimedia Appendix 6).

Beyond technical validation, the findings demonstrated practical value for clinical research operations. Large-scale trials and observational studies often face delays in cohort assembly and iterative validation; Parquet-based pipelines shorten these steps, enabling more adaptive study designs. In multicenter collaborations, they promote secure and efficient data exchange, reducing delays that commonly impede cooperative studies.

### Limitations

Several limitations should be acknowledged. First, the datasets analyzed were derived from specific institutional cohorts, which may constrain generalizability to other health care systems. Second, the elevated performance baseline of the predictive models limited the sensitivity to subtle differences between CSV and Parquet formats. Third, while MIA offered a widely accepted proxy for privacy risk, broader adversarial evaluations were not included. Fourth, the evaluation was primarily conducted in on-premise settings, so the scalability of findings in distributed or cloud-based environments remains to be established. Finally, despite the clear efficiency advantages of Parquet, its adoption may introduce a technical learning curve for clinical researchers who are less familiar with column-oriented storage formats or big-data frameworks. Transitioning from traditional row-oriented workflows to optimized columnar architectures requires initial investments in technical training and infrastructure adjustment. However, these upfront efforts are likely to be offset by the long-term gains in scalability and processing speed demonstrated in our results, ultimately supporting more efficient and reproducible clinical evidence generation.

### Future Work

Future research should extend the evaluation of Parquet-based pipelines to distributed and cloud-based environments, focusing on both scalability and cost-effectiveness. In addition, validation across hybrid and federated architectures will be important to confirm applicability in multicenter collaborations where data cannot be centralized. Beyond structured EHR data, future studies should also investigate applications to unstructured modalities, such as clinical narratives, imaging metadata, and time-series signals. Such efforts would test the versatility of Parquet-based pipelines in supporting multimodal learning and integrative analyses. Privacy assessments should likewise be expanded to encompass attribute inference, linkage, and other adversarial scenarios, ideally within federated or privacy-preserving analytic frameworks. Moreover, interoperability across international coding standards should be validated using external multicenter datasets to ensure global applicability. Pursuing these directions will enhance the technical robustness of Parquet-based infrastructures while reinforcing their clinical relevance, ultimately enabling secure collaboration and more efficient integration of evidence into patient care.

### Conclusions

This study demonstrated that Parquet-based end-to-end processing effectively alleviated the storage and computational bottlenecks inherent in traditional row-oriented and relational database workflows while maintaining predictive fidelity and privacy safeguards. By improving computational efficiency and enabling the rapid preparation of analysis-ready cohorts, Parquet reduces the resources required for large-scale clinical data handling. These efficiencies translate into tangible research benefits, enabling earlier study initiation and more adaptive study designs across diverse clinical research settings.

The integration of efficiency with privacy safeguards further ensures that accelerated workflows remain suitable for secure data sharing across institutions. In parallel, these capabilities support regulatory-grade analyses and health technology assessments, where timely and reproducible evidence is critical. For clinicians and researchers, the central implication is that Parquet-based pipelines reduce the manual effort and computational overhead required to transform raw EHR data into analysis-ready cohorts. This capability not only strengthens multicenter collaboration but also facilitates the timely translation of evidence into clinical decision support.

Ultimately, by aligning computational efficiency with privacy-conscious infrastructure, Parquet-based pipelines reinforce the evidence base that informs clinical decision-making, positioning this approach as a robust foundation for sustainable precision medicine.

## Appendix

Multimedia Appendix 1 Column definitions for the structured clinical data table.

Multimedia Appendix 2 Synthetic example data used for scenario-based workload evaluation.

Multimedia Appendix 3 Resource use profiles during Parquet-based data processing across different chunk sizes.

Multimedia Appendix 4 Hyperparameter configurations and the formal statistical framework for equivalence testing.

Multimedia Appendix 5 Detailed demographic characteristics and visit statistics of the study cohort.

Multimedia Appendix 6 Calibration metrics (Brier score and expected calibration error [ECE] and ECE) for each binary classification task.

Multimedia Appendix 7 Integrated statistical performance and equivalence analysis.

Multimedia Appendix 8 Membership inference results with shadow and Likelihood Ratio Attack attackers.

## Abbreviations

AI: artificial intelligence
AUC: area under the curve
AUPRC: area under the precision-recall curve
AUROC: area under the receiver operating characteristic curve
CC: classifier chains
CPU: central processing unit
ECE: expected calibration error
EHR: electronic health record
GPU: graphics processing unit
ICD-10: International Classification of Diseases, Tenth Revision
KCD-8: Korean Classification of Diseases, Eighth Revision
LiRA: Likelihood Ratio Attack
MIA: membership inference attack
OvR: one-vs-rest
TOST: two 1-sided tests
XGBoost: extreme gradient boosting

## Data Availability

The datasets generated or analyzed during this study are available from the corresponding author on reasonable request.
