# Supplementary material for: Scalable and Privacy-Conscious End-to-End Processing of Large-Scale Clinical Data for Precision Medicine: Empirical Evaluation Study
Source: JMIR Med Inform. 2026 Mar 4;14:e83487. doi: 10.2196/83487 (PMC13000379; doi:10.2196/83487)

Table S1**.** Processing efficiency by chunk size.

| Chunk | Duration, s | Throughput, rows/s | CPU^a^, % | Memory^b^, GB | I/O^c^ read, s | I/O write, s |
| --- | --- | --- | --- | --- | --- | --- |
| 10,000 | 362.0 | 37,051 | 54.3 | 10.1 | 60.0 | 108.8 |
| 25,000 | 346.4 | 38,692 | 59.0 | 9.0 | 63.0 | 85.7 |
| 50,000 | 351.4 | 38,151 | 57.6 | 9.3 | 74.8 | 84.1 |

^a^CPU: CPU utilization.

^b^Memory: Memory usage during processing.

^c^I/O: Disk access time, representing the total duration for data read and write operations.

Figure S1. Resource utilization profiles during Parquet-based data processing across different chunk sizes.


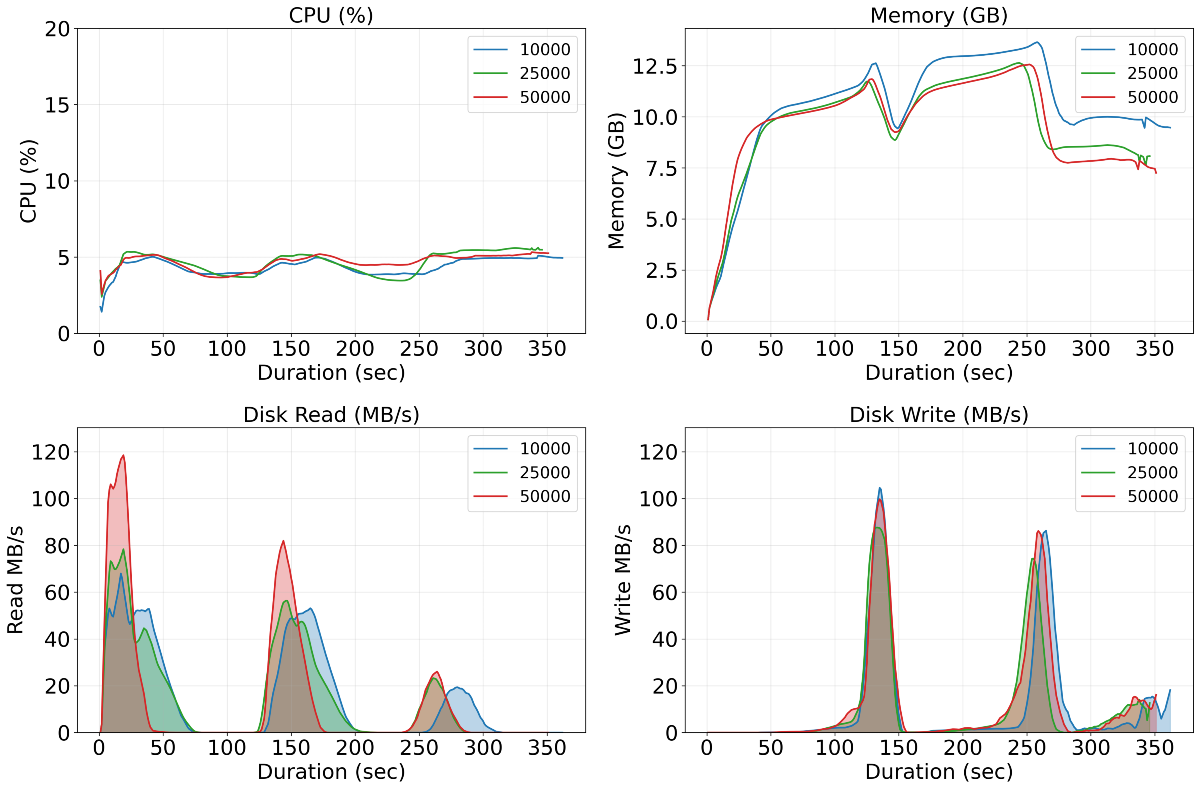

Supplement: Multimedia Appendix 3 [file medinform_v14i1e83487_app3.docx]
