# Supplementary material for: Scalable and Privacy-Conscious End-to-End Processing of Large-Scale Clinical Data for Precision Medicine: Empirical Evaluation Study
Source: JMIR Med Inform. 2026 Mar 4;14:e83487. doi: 10.2196/83487 (PMC13000379; doi:10.2196/83487)
Supplement: Multimedia Appendix 4 [file medinform_v14i1e83487_app4.docx]

Table S1. Hyperparameters and configurations for XGBoost and multi-label strategy.

| Category | Configuration Parameter | Value |
| --- | --- | --- |
| **Model Engine** |  |  |
|  | Library version and name | XGBoost 2.0.3 |
|  | Computation platform | GPU-accelerated |
|  | Tree algorithm | gpu_hist |
|  | Objective function | binary:logistic |
| **XGBoost** |  |  |
|  | Learning rate (eta) | 0.08 |
|  | Maximum depth | 6 |
|  | Subsample rate | 0.8 |
|  | Column subsample rate | 0.8 |
|  | n_estimators | 1,000 |
|  | Early stopping rounds | 50 |
|  | Imbalance handling | scale_pos_weight$^a$ |
| **Multi-label Strategy** |  |  |
|  | Modeling approach | Classifier Chains (CC) and One-vs-Rest (OvR) |
|  | CC Ensemble count | 5 |
|  | Aggregation method | Arithmetic mean |
| **Reproducibility** |  |  |
|  | Random seed, n | 42 |
|  | Data partitioning, Train | 65% |
|  | Data partitioning, Validation | 15% |
|  | Data partitioning, Test | 20% |

Table S2. Statistical analysis settings for performance evaluation and equivalence testing.

| Category | Parameter | Value |
| --- | --- | --- |
| **Experimental Design** | Independent experimental runs, n | 20 |
| **Statistical Robustness** |  |  |
|  | Bootstrap resampling iterations, n | 5,000 |
|  | Confidence interval (CI), % | 95 |
| **Equivalence Testing** |  |  |
|  | Equivalence margin for AUROC and AUPRC, δ | 0.02 |
|  | Equivalence margin for accuracy and F1-score, δ | 0.01 |
| **Significance Testing** |  |  |
|  | Alpha level for equivalence testing (TOST) , *P*-value | 0.05 |
|  | Alpha level for paired tests (Bonferroni-adjusted) , *P*-value | 0.001 |
