# Supplementary figures and images for: Scalable and Privacy-Conscious End-to-End Processing of Large-Scale Clinical Data for Precision Medicine: Empirical Evaluation Study
Source: JMIR Med Inform. 2026 Mar 4;14:e83487. doi: 10.2196/83487 (PMC13000379; doi:10.2196/83487)

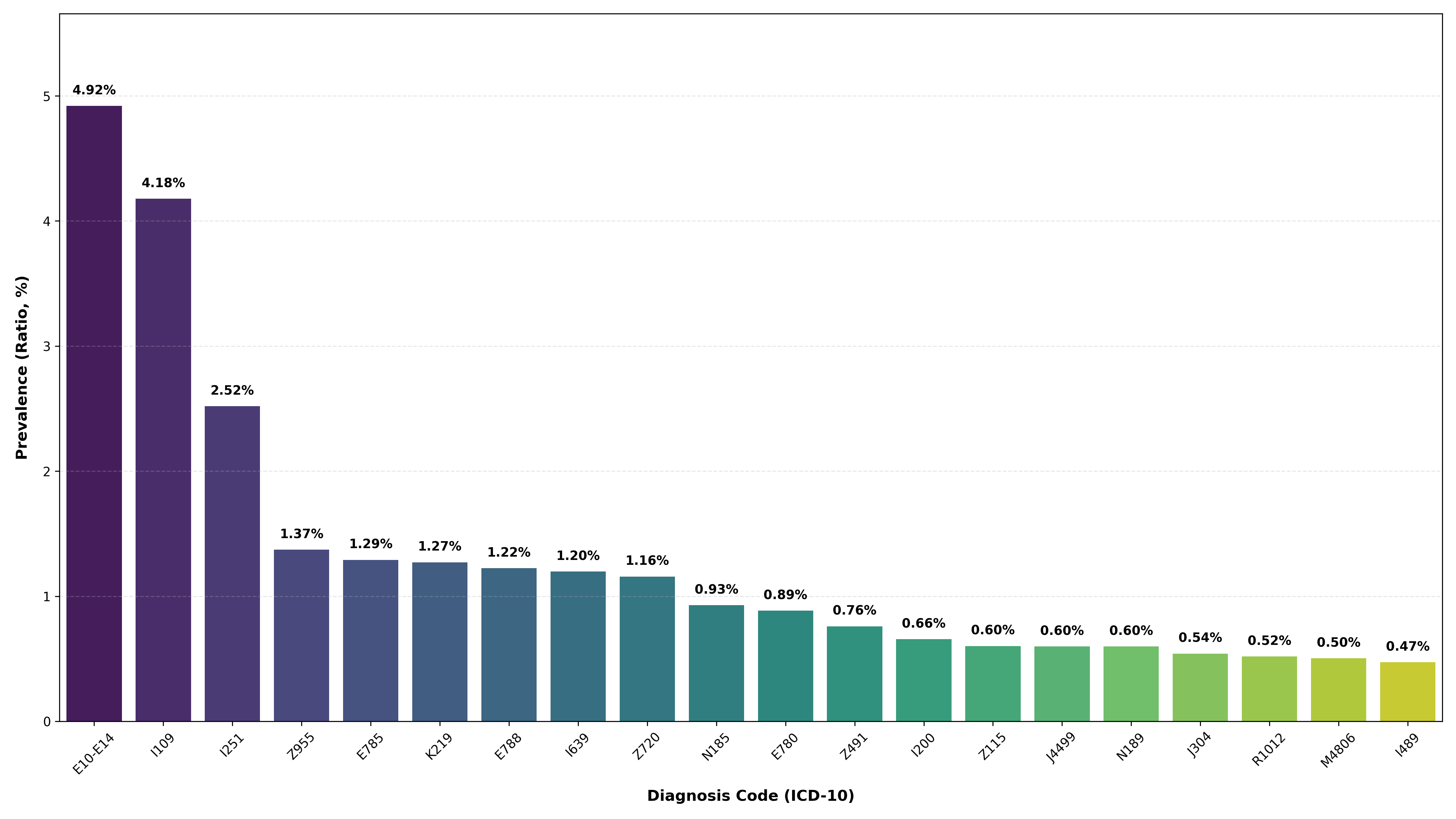

Supplement: Multimedia Appendix 5 [file medinform_v14i1e83487_app5.png]
