# Supplementary material for: Scalable and Privacy-Conscious End-to-End Processing of Large-Scale Clinical Data for Precision Medicine: Empirical Evaluation Study
Source: JMIR Med Inform. 2026 Mar 4;14:e83487. doi: 10.2196/83487 (PMC13000379; doi:10.2196/83487)
Supplement: Multimedia Appendix 6 [file medinform_v14i1e83487_app6.docx]

Table S1. **Calibration metrics (Brier score and Expected Calibration Error, ECE) for each binary classification task.**

| Metric | Hypertension | Spine | Diabetes | Macro mean |
| --- | --- | --- | --- | --- |
| Brier score | 2.1×10⁻⁵ | 0.000 | 0.000 | 6.9×10⁻⁶ |
| ECE | 6.0×10⁻⁶ | 0.016 | 0.018 | 0.019 |

**Brier score: Quantifies the squared error between predicted probabilities and true labels.**

**ECE: Expected calibration error; measures the mismatch between predicted confidence and observed accuracy.**
