# Supplementary material for: Scalable and Privacy-Conscious End-to-End Processing of Large-Scale Clinical Data for Precision Medicine: Empirical Evaluation Study
Source: JMIR Med Inform. 2026 Mar 4;14:e83487. doi: 10.2196/83487 (PMC13000379; doi:10.2196/83487)
Supplement: Multimedia Appendix 7 [file medinform_v14i1e83487_app7.docx]

Table S1. Classification performance comparison between CSV and Parquet conditions.

| Metric | CSV median (IQR ^a^) | Parquet median (IQR) | Difference^b^ | *P* value |
| --- | --- | --- | --- | --- |
| AUROC^c^ | 0.985 (0.985-0.985) | 0.984 (0.984-0.984) | -1.00 × 10⁻³ | < .001 |
| AUPRC^d^ | 1.000 (1.000-1.000) | 1.000 (1.000-1.000) | 0.000 | < .001 |
| Micro F1 | 1.000 (1.000-1.000) | 1.000 (1.000-1.000) | 0.000 | < .001 |
| Macro F1 | 1.000 (1.000-1.000) | 1.000 (1.000-1.000) | 0.000 | < .001 |
| Subset accuracy | 1.000 (1.000-1.000) | 1.000 (1.000-1.000) | 0.000 | < .001 |

^a^**IQR: Interquartile range.**

^b^**Difference:** Parquet median – CSV median.

^c^**AUROC: Area under the receiver characteristic curve.**

^d^**AUPRC: Area under the precision-recall curve.**

Table S2. Classification performance comparison between CSV and Parquet conditions using paired tests.

| Metric | CSV mean | Parquet mean | Difference^a^ | Paired t-test^b^ *P* value | Wilcoxon^c^ *P* value |
| --- | --- | --- | --- | --- | --- |
| AUROC | 1.000 | 0.984 | -1.51 × 10⁻² | < .001 | < .001 |
| AUPRC | 1.000 | 0.999 | -5.00 × 10⁻⁷ | < .001 | < .001 |
| Accuracy | 1.000 | 0.999 | -6.90 × 10⁻⁶ | < .001 | < .001 |
| F1-score | 1.000 | 0.999 | -5.20 × 10⁻⁶ | < .001 | < .001 |

^a^Difference: Parquet mean – CSV mean.

^b^Paired t-tests consistently indicated significant differences (Bonferroni-adjusted P < .001).

^c^Wilcoxon signed-rank tests consistently indicated significant differences (Bonferroni-adjusted P < .001).

Table S3**. Equivalence testing (TOST) and bootstrap confidence intervals for classification performance differences between CSV and Parquet conditions.**

| Metric | Difference^a^ | Equivalence margin, **δ** | TOST Equivalence^b^ | Bootstrap 95% CI |
| --- | --- | --- | --- | --- |
| AUROC | −1.51 × 10⁻² | 0.02 | Yes | (−1.56 × 10⁻², −1.50 × 10⁻²) |
| AUPRC | −1.00 × 10⁻⁶ | 0.02 | Yes | (−1.20 × 10⁻⁶, −0.97 × 10⁻⁶) |
| Accuracy | −7.00 × 10⁻⁶ | 0.01 | Yes | (−7.21 × 10⁻⁶, −6.85 × 10⁻⁶) |
| F1-score | −5.00 × 10⁻⁶ | 0.01 | Yes | (−5.60 × 10⁻⁶, −4.89 × 10⁻⁶) |

^a^Difference: Parquet mean – CSV mean.

^b^**TOST: Two one-sided tests statistical equivalence was confirmed at the *P* < .05 level based on the pre-specified margins (δ = 0.02 or 0.01).**
