# Supplementary material for: Scalable and Privacy-Conscious End-to-End Processing of Large-Scale Clinical Data for Precision Medicine: Empirical Evaluation Study
Source: JMIR Med Inform. 2026 Mar 4;14:e83487. doi: 10.2196/83487 (PMC13000379; doi:10.2196/83487)
Supplement: Multimedia Appendix 8 [file medinform_v14i1e83487_app8.docx]

Table S1. Supplementary membership-inference results with Shadow and LiRA attackers.

| Method | Attack AUC^a^ | Bootstrap 95% CI^b^ | Advantage^c^ | Best accuracy^d^ | Gain vs baseline^e^ |
| --- | --- | --- | --- | --- | --- |
| Shadow | 0.502 | (0.501, 0.502) | 0.003 | 0.502 | 0.002 |
| LiRA | 0.506 | (0.504, 0.508] | 0.003 | 0.501 | 0.001 |

^a^**Attack AUC: Denotes area under the ROC curve.**

^b^**Bootstrap 95% Cis for the AUC were computed using the bootstrap resampling. For LiRA, the CI reflects the evaluated sample distribution (~300,000, members/non-members).**

^c^**Advantage = max(TPR − FPR).**

^d^**Best accuracy = max{(TPR + (1 − FPR))/2}.**

^e^**Gain vs Baseline = Best accuracy − 0.500.**
